# Re-irradiation of recurrent head and neck carcinomas: comparison of robust intensity modulated proton therapy treatment plans with helical tomotherapy

**DOI:** 10.1186/1748-717X-8-93

**Published:** 2013-04-20

**Authors:** Martin Stuschke, Andreas Kaiser, Jehad Abu-Jawad, Christoph Pöttgen, Sabine Levegrün, Jonathan Farr

**Affiliations:** 1 Department of Radiotherapy, University Duisburg-Essen, 45147 Essen, Germany; 2 Westdeutsches Protonentherapiezentrum Essen, 45147 Essen, Germany; 3 Current address: Department of Radiologic Sciences, St. Jude Children's Research Hospital, Memphis, TN, USA

**Keywords:** IMPT, Intensity modulated proton therapy, Robust optimization, Robustness, Plan comparison, Re-irradiation, Helical tomotherapy

## Abstract

**Background:**

To test the hypothesis that the therapeutic ratio of intensity-modulated photon therapy using helical tomotherapy (HT) for retreatment of head and neck carcinomas can be improved by robust intensity-modulated proton therapy (IMPT).

**Methods:**

Comparative dose planning with robust IMPT was performed for 7 patients retreated with HT.

**Results:**

On average, HT yielded dose gradients steeper in a distance ≤ 7.5 mm outside the target (p<0.0001, F-test) and more conformal high dose regions down to the 50% isodose than IMPT. Both methods proved comparably robust against set-up errors of up to 2 mm, and normal tissue exposure was satisfactory. The mean body dose was smaller with IMPT.

**Conclusions:**

IMPT was found not to be uniformly superior to HT and the steeper average dose fall-off around the target volume is an argument pro HT under the methodological implementations used. However, looking at single organs at risk, the normal tissue sparing of IMPT can surpass tomotherapy for an individual patient. Therefore, comparative dose planning is recommended, if both methods are available.

## Background

Re-irradiation of late recurrent head and neck cancer with concomitant chemotherapy is a curative option for un-resectable recurrences. However, side effects are significant. Randomized or prospective uncontrolled trials with total radiation doses of 60–65 Gy at 1.5 - 2.0 Gy per fraction with or without concomitant chemotherapy showed a crude incidence of late grade III or worse side effects of 34% - 65% [1-4]. For patients with non-nasopharyngeal head and neck carcinomas treated within these trials, the predominant late side effects were trismus, osteoradionecrosis, subcutaneous fibrosis, late mucosal side effects, and pharyngeal, laryngeal, esophageal dysfunctions or carotid ruptures. Highly conformal techniques are used frequently. In their large retrospective series, Lee et al. [5] found a higher freedom from loco-regional progression at 2 years with intensity modulated photon radiotherapy techniques in comparison to 3D conformal radiotherapy, indicating that the therapeutic index in the retreatment of head and neck cancer can be improved with more conformal techniques.

Recent reviews on proton radiotherapy for head and neck cancer concluded that protons are able to improve the therapeutic ratio by significantly decreasing normal tissue dose, while keeping similar or better target coverage than current photon techniques, and that scanned intensity modulated proton therapy (IMPT) might prove most advantageous [6,7].

In this study, we review a series of re-irradiated patients with long term follow-up, treated with helical tomotherapy (HT). We conducted comparative intensity modulated proton therapy planning using an advanced robust optimization algorithm. The planning aim was to create proton plans reaching or surpassing the good target volume coverage achieved with HT while offering better sparing of the surrounding normal tissues.

## Methods

For comparative planning with IMPT, 7 patients with recurrent carcinoma in the head and neck were identified, who had received a radical retreatment with HT between January 2009 and September 2010. Median and minimum time between the start of the first and second treatment series was 48 months and 37 months, respectively, allowing partial recovery of potential damage in the CNS [8]. The total radiation dose, given in the first radiotherapy series, was 56 – 63 Gy with conventional fractionation. All patients were immobilized in a frameless precision head mask system developed for fractionated stereotactic treatments (BrainLAB AG, Feldkirchen, Germany).

Planning computed tomograms (CT) were obtained using a large-bore CT scanner (Somatom Sensation Open, Siemens, Erlangen, Germany). CT slices of 1.5 - 2.0 mm thickness were reconstructed. A CTV margin of 0.5 - 1.0 cm was applied around the GTV, respecting non-involved anatomic borders. An isotropic PTV margin of 0.2 cm was added to the CTV for the HT treatment planning. A constant relative biological effectiveness (RBE) of 1.0 was assumed for photons and of 1.1 for protons.

### Tomotherapy treatment planning

HT plans were normalized such that 95% of the PTV was covered by the prescribed dose in 5 patients and 90% of the PTV in two patients. Planning parameter combinations used were field width (FW) of 1.0 cm with a pitch of 0.215 (4 patients), FW 1.0 cm with pitch 0.287 (1 patient), and FW 2.5 cm with pitch 0.215 (2 patients), respectively, depending on the cranio-caudal extent of the target volume. The dose calculation grid had a size of 1.95 mm × 1.95 mm in the axial plane. Modulation factors between 1.6 and 2.4 were obtained. Dose constraints differed from patient to patient due to the individual nature of re-irradiation. The planning goal was to keep the lifetime dose to the spinal cord and brain stem surface dose below 60 Gy and 64 Gy, respectively [8]. Two patients, patients 4 and 6, were treated by an integrated boost technique, increasing the dose to the boost PTV by 25% and 18.5% in comparison to the larger PTV1, respectively. All treatments were given in a radical intent. The biologically equivalent dose (BED) to the tumor was calculated according to Ho et al. [9], assuming a fractionation sensitivity of the tumor characterized by an α/β ratio of 10 Gy, a time delay to onset of compensatory repopulation of 21 d, and a repopulation rate of 0.66 Gy/d thereafter. The BED for an individual patient was expressed as the total dose of a conventional fraction schedule at 5×2 Gy / week resulting in the same BED (2 Gy/fraction equivalent scheme). The intended total radiation dose was equivalent to ≥ 60 Gy with 2 Gy/d fraction scheme for each patient, the recommended dose range for re-irradiation of patients with a response duration of ≥ 6 months according to the American College of Radiology and the National Comprehensive Cancer Network Guidelines [10,11].

### Proton therapy planning

Comparative IMPT planning was done using a RayStation v2.4.13.31 system, developed by RaySearch Laboratories within a Partnership with the WPE. Robust IMPT plans were obtained applying a minimax optimization to account for range- as well as setup-uncertainties [12]. The optimizer aims at minimizing the objective of the worst case scenario. Set-up errors were simulated by moving spot weights to corresponding adjacent spot positions within the iso-energy layers for each beam. Density errors were approximated by calculating dose for several density scalings. For IMPT, set-up errors of 2 mm were considered as well as range uncertainties of ±3.5%. We investigated potentials of robust IMPT to create plans superior to HT. Optimization criteria for IMPT ordered according to importance were: (1) Robust CTV coverage at least as good as with HT, (2) D1cc to the brain stem or spinal cord equal or smaller, (3) D2 within the ipsilateral optic nerve or the chiasma opticum equal or smaller, (4) D2< 115% within the CTV, (5) D1cc (D5cc) as well as Dmean for the ipsilateral temporal lobe (cerebellum) smaller, (6) conformity around the CTV higher, (7) Dmean to the parotid glands smaller, and (8) V80 of the lower jaw adjacent to the tumor smaller than in the HT plan.

Quantitative Analysis of Normal Tissue Effects in the Clinic (QUANTEC) review states Dmax as appropriate dose-volume histogram parameter to assess side effect probabilities in most CNS structures [13]. But as most of the underlying empirical data was derived from studies before the IMXT era, we used the D2 criterion or, if empirically established, the D1cc - D5cc parameters for the brain, temporal lobes and brain stem, respectively, to restrict the high dose area in the CNS during optimization [13,14].

The beam model used for IMPT optimization is made for the IBA dedicated pencil beam nozzle. The proton pencil beam sigma values were 3.66 and 3.00 mm in air at isocenter at beam energies of 150 and 230 MeV, respectively. Energies below 100 MeV were not used. Additional range shifters were introduced as appropriate to cover the target volume proximally. The proton beam scanning grid was 5 mm in both orthogonal directions within an energy layer and the distance between iso-energy layers in water was 5 mm.

Multiple field IMPT plans were used for all patients. A 4 fields set up was chosen for patients 3, 5, 6, and 7. A 5, 6, and 7 fields set up was used for patients 2, 4 and 1, respectively. For patient 1, the near central PTV neighboring the brainstem was tangentially approximated by 3 bi-lateral fields, as well as by a p-a field. Thus, the optimizer had the freedom to increase spot weights in tangential fields at the CTV surfaces near critical organs at risk, i.e. the spinal cord, brain stem, temporal lobes and cerebellum, and to avoid spots stopping right in front of these organs. For more lateralised target volumes, as in patients 2–7, two or three ipsi-lateral fields were used with field normals tangential to the major parts of the anterior and posterior surfaces of the target volume. Gantry angels between these fields ranged from 50° to 100°. For most patients, one additional field with an intermediary gantry angle between those fields was used. Furthermore, one field from the near ap- or pa direction or alternatively a contra-lateral field was used to cover medial parts of the target volume without exposure of the spinal cord or brain stem. Dose conformity was measured by the Paddick conformity index CI_X%_ = V_x_*V_x_/(BV_X_*CTV), where V_x_ is the intersection volume between the CTV and BV_X_, the body volume receiving x% of the prescribed dose. The CTV was used as reference because the planning aim of both, HT and IMPT plans was to cover the CTV robustly, but IMPT plan robustness did not rely on a PTV. To estimate mean dose gradients outside the PTV, 8 adjacent shells of 1 mm width were constructed around each PTV1 within the body volume by isotropic expansion of the PTV1. For estimation of the dose fall off outside the PTV, the mean dose or the D90 in each shell was plotted against the mid-distance of the respective shell from PTV.

### Statistical analysis

Pair-wise comparisons of IMPT and HT according to one parameter from the dose volume histogram (DVH) of an organ at risk or target volume were performed for all patients using the signed rank test, Proc Univariate, SAS statistical software Version 9.2 (Cary, NC). Regression analyses were performed using the Proc GLM from SAS.

## Results

**Figure 1:**
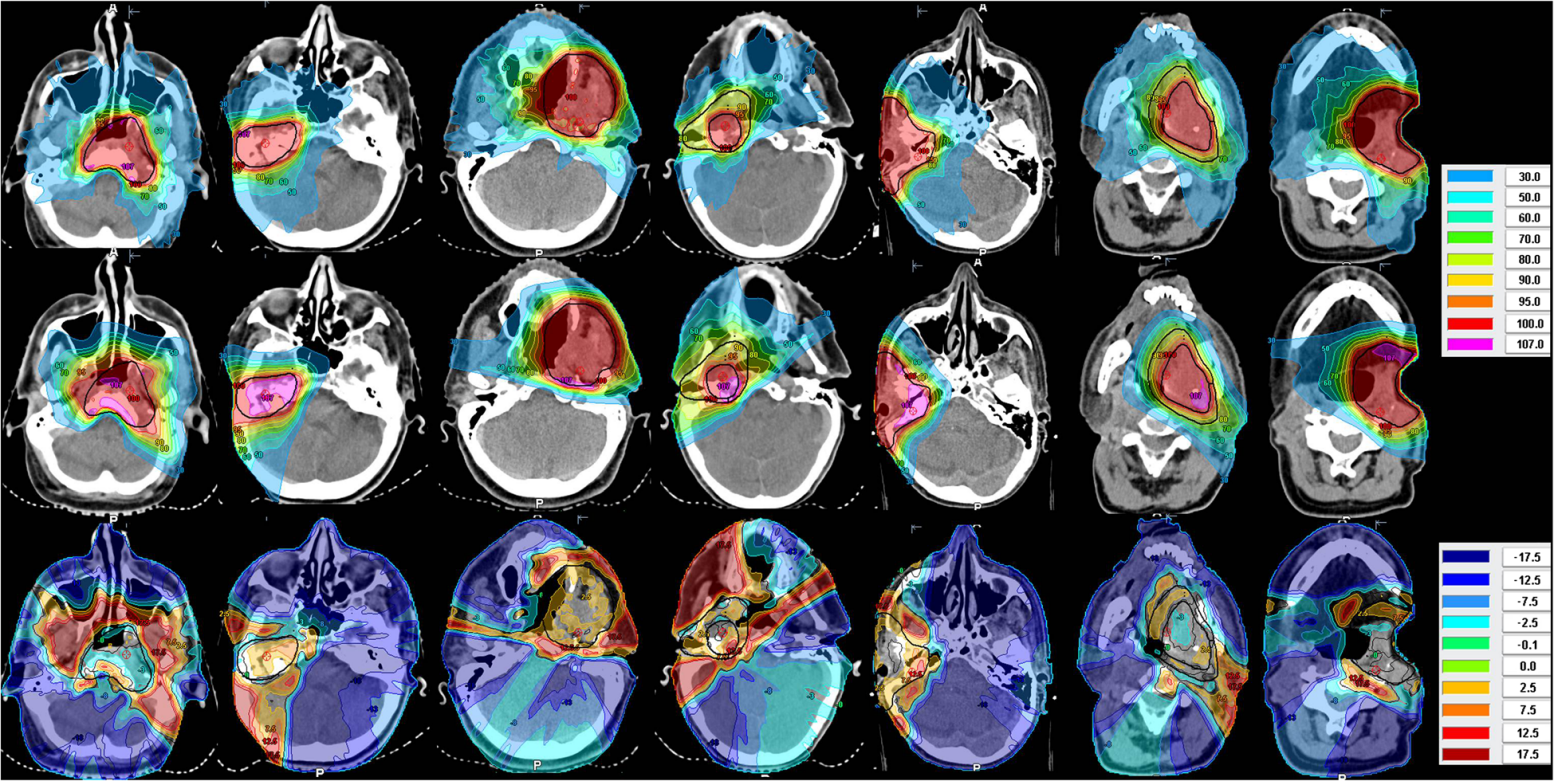
**Dose distribution.** Dose distributions for patients 1–7 from left to right. Helical tomotherapy (HT) and intensity modulated proton therapy (IMPT) plans are shown in row 1 and row 2. Corresponding dose difference plots (IMPT minus HT) are given in the third row for the respective patients.

Patients 2, 4, 5 had a recurrent carcinoma of the auditory canal, while patient 7, and 3 had a recurrent oropharyngeal, patient 1 a nasopharyngeal, and patient 6 a floor of mouth carcinoma. The received re-irradiation total 2Gy/fraction equivalent dose was 56 Gy - 68 Gy. Five patients received Cisplatin at 30 mg/m^2^ weekly, the remaining two refused chemotherapy. With respect to clinical outcome, no patient experienced grade 3 late toxicities. Median follow-up of the seven patients is 39 (31–48) months. Survival at 36 months is 57%. Figure 1 illustrates dose distributions achieved with HT and IMPT in the first and second row, respectively. Dose difference plots are given in the third row.

### Coverage of the CTV

For HT and IMPT, the CTV D95 was equal or larger than the prescribed dose for all patients except patient 1, in whom it was ≥ 97.5% of the prescribed dose (Table 1). In this patient, the smallest distance between CTV and brainstem fell below 2 mm so that robustness against set-up errors and fulfillment of strict brain stem were competitive objectives. Patients 4 and 6 received an integrated boost, and their plans were normalized to the prescribed dose in the CTV_boost_. The prescribed doses to the larger CTV1 were 84.1% and 80.0% of the prescribed CTV_boost_ dose, respectively. Robustness of the IMPT and HT plans was evaluated within scenarios 1–5, applying a translation of the isocenter of the beam arrangement against the image data set of −2.0/2.0/-2.0/2.0/1.5 mm in the ventral, -2.0/2.0/2.0/-2.0/-1.5 mm in the ipsilateral, and 0.0/ 0.0/ 0.0/0.0/1.5 mm in the cranial direction. In addition, density changes by +3%, and −3% were applied to the IMPT plans in scenario 5. The CTV D95 was found stable within 4% and did not fall below 98% of the prescribed dose in 6 of the 7 patients for both IMPT and HT. In patient 1 however, D95 decreased to 85.5% for HT and to 89.1% for IMPT. A pairwise comparison of the D95 values of IMPT and HT over all the set-up errors tested did not result in significant robustness differences between the plans for both radiotherapy methods (p=0.13, F-test). The D2 values for brainstem or spinal cord increased over the scenarios on average by 3.7% (range, -9.4% - +26%) of the prescribed dose over the different patients for IMPT and by 3.1% (range, -8.3% - +19%) for HT with no significant difference between HT and IMPT (p=0.58, F-test). Unique to IMPT, density changes of +3% and −3% within the set-up scenario 5 led to maximum increases of brain stem D2 by 19% and 15% of the prescription dose in patients 1 and 2, respectively, and stayed constant within 4% in the other patients in comparison to scenario 5 without density changes. The D95 for the CTV remained constant within 2% of the prescribed dose for all patients except patient 1, where it decreased by up to 6.7%.

**Table 1:** Dose volume parameters

	**Patient 1**	**Patient 2**	**Patient 3**	**Patient 4**	**Patient 5**	**Patient 6**	**Patient 7**
	**Proton / Photon**	**Proton / Photon**	**Proton / Photon**	**Proton / Photon**	**Proton / Photon**	**Proton / Photon**	**Proton / Photon**
**CTV**	60	33	97	71	127	197	115
				11		66	
Paddick CI95	0.83 / 0.93	0.46 / 0.58	0.57 / 0.66	0.46 / 0.71	0.69 / 0.79	0.78 / 0.74	0.69 / 0.71
				0.21 / 0.46		0.58 / 0.64	
D98	86.5 / 91.2	100.6 / 102.0	100.9 / 99.4	86.2 / 85.7	99.9 / 99.9	79.0 / 82.1	100.4 / 101.8
				101.3 / 100.5		104.7 / 101.3	
D95	97.5 / 98.5	102.0 / 102.3	101.1 / 99.7	88.4 / 85.5	101.2 / 100.5	80.9 / 82.8	100.4 / 102.1
				101.4 / 100.8		101.2 / 103.2	
D50	104.4 / 105.3	105.3 / 103.8	103.8 / 101.3	95.6 / 89.9	105.2 / 102.6	91.6 / 88.2	104.0 / 103.3
				104.2 / 102.0		104.7 / 104.7	
D2-D98	30.3 / 16.5	13.5 / 4.1	9.2 / 4.2	26.6 / 15.9	12.2 / 5.0	24.5 / 20.8	9.9 /3.4
				16.2 / 3.2		8.4 / 3.4	
**Spinal Cord**							
D2	8.7 / 4.8	9.2 / 14.4	2.6 / 9.7	4.7 / 5.9	5.3 / 13.2	8.5 / 8.7	13,7 / 19.3
Dmax	16.6 / 8.2	13.5 / 18.7	10.2 / 16.0	14.4 / 13.7	13.3 / 18.7	15.4 / 13.3	25.0 / 28.1
**Brain stem**							
D1cc	18.8 /21.6	41.0 / 45.0	11.6 / 11.9	14.3 / 16.5	30.6 / 46.5	3.8 / 5.0	4.7 / 6.2
Dmax	38.5 /40.0	79.5 / 78.7	30.1 / 22.7	33.5 / 25.0	61.6 / 64.5	8.2 / 9.0	13.6 / 10.6
**ipsilat. Optic Nerve**							
Dmax	32.5 / 13.8	22.6 / 26.1	11.9 / 3.8	3.9 / 2.4	2.8 / 18.6	0.0 / 1.6	0.0 / 1.2
**Chiasma Opticum**							
Dmax	18.7 / 15.4	28.1 / 15.1	0.8 / 3.4	6.3 / 2.7	2.0 / 16.0	0.0 / 1.4	0.0 / 1.2
**Temporal Lobes**							
ipsilat. Dmean	17.9 / 21.9	54.4 / 50.9	5.9 / 10.7	28.6 / 15.5	47.3 / 57.1	0.3 / 1.6	0.7 / 1.2
ipsilat. D1cc	72.5 / 77.7	98.3 / 99.8	32.0 / 34.6	86.1 / 76.1	106.8 / 100.8	2.8 / 3.1	0.1 / 2.1
**contoured Cerebellum**							
Dmean	9.0 / 18.4	13.3 / 26.2	0.5 / 9.8	9.1 / 16.4	11.1 / 32.1	0.2 / 2.5	0.1 / 1.4
D5cc	53.9 / 53.7	69.7 / 60.7	3.7 / 21.7	52.0 / 47.2	74.1 / 67.9	2.0 / 9.4	1.2 / 2.8
**Parotid Glands**							
Dmean	37.3 / 26.9	3.0 / 4.5	17.6 / 33.0	1.2 / 7.9	0.0 / 4.8	24.3 /39.0	48.7 / 47.8
**contoured Jaw**							
V80	0.6 / 0.1	4.1 / 2.2	11.2 / 11.5	16.6 / 10.5	7.8 / 6.7	5.7 / 6.0	9.3 / 10.1
Dmean	13.0 / 7.4	8.6 / 8.4	20.9 / 24.4	35.9 / 34.3	12.3 / 15.6	34.2 / 48.0	25.4 / 39.4
**scanned Body**							
Dmean	10.2 / 11.1	6.9 / 9.5	7.2 / 9.7	4.4 / 4.4	3.2 / 5.7	3.7 / 5.3	3.6 / 4.9
V95	89 / 83	73 / 57	171 / 147	156 / 100	186 / 161	254 / 266	167 / 162
V90	108 / 92	87 / 64	193 / 167	178 / 112	205 / 177	281 / 291	190 / 180
V80	143 / 112	114 / 77	234 / 200	221 / 132	247 / 207	337 / 343	231 / 212
**PTV**	81	49	124	92	152	246	147
				17		92	

Dose volume parameters related to target coverage, homogeneity and the exposure of normal tissues by helical tomotherapy (Photon) and intensity modulated proton therapy (Proton). All doses were given in percent of the prescribed dose, all volumes in cm^3^. Patients 4 and 6 were treated by an integrated boost technique and their plans were normalized to the prescribed boost dose. For these two patients, the respective cells for the D2 to D98 values were split and the values for the boost clinical target volume (CTV) were given in the lower half and for the larger CTV1 in the upper half of the cell.

### Conformity

**Figure 2:**
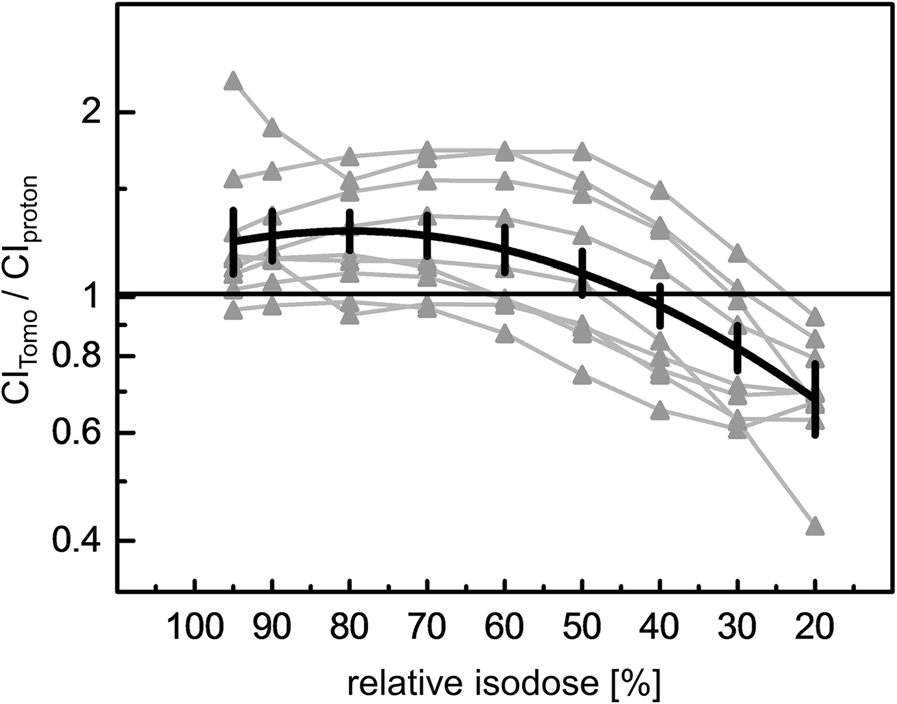
**Conformity indices.** Ratio of the Paddick conformity indices for helical tomotherapy (CItomo) and intensity modulated proton therapy (CIproton) plans for all target volumes of this study. The ratios are given for isodose values from 95% to 20% of the prescribed dose. Data points related to the same target volume are connected by grey lines. The average dependence of the conformity index ratio on isodose was estimated by linear quadratic Taylor series expansion (black solid curve). The 95% confidence intervals of the predicted mean isodoses by this fit are drawn as black vertical bars.

The median CTV volume was 86 cm^3^ (Table 1). The Paddick conformity indices (CI) at the 95% isodose were larger for the HT plans in comparison to robust IMPT in 8 of the 9 target volume comparisons, including the boost volumes given in patients 4 and 6 (p=0.04, signed rank test). Figure 2 shows the Paddick CI_Tomo_/ CI_proton_ ratio for 9 target volumes of the evaluated patients at different isodose values between 95% and 20%. The drawn line is a linear quadratic fit as Taylor series expansion to the last significant term of this ratio on the isodose level. The predicted mean value of CI_Tomo_/CI_proton_ and its 95% confidence interval ratio from this fit was >1 from the 95% isodose down to the 50% isodose, indicating considerably higher average conformity for HT compared with IMPT at these higher isodose levels, while robust IMPT was more conformal for the 30% and lower isodoses. The shallower average dose fall off in the IMPT-plans is visualized as a red shell around the CTV in the dose differences plots of Figure 1, third row. In addition, we looked at the effect of reducing the range uncertainties during optimization from ±3% to ±1.5% on CI95 of the IMPT plans. The Paddick CI95% was found stable within ±2% without a systematic increase.

**Figure 3:**
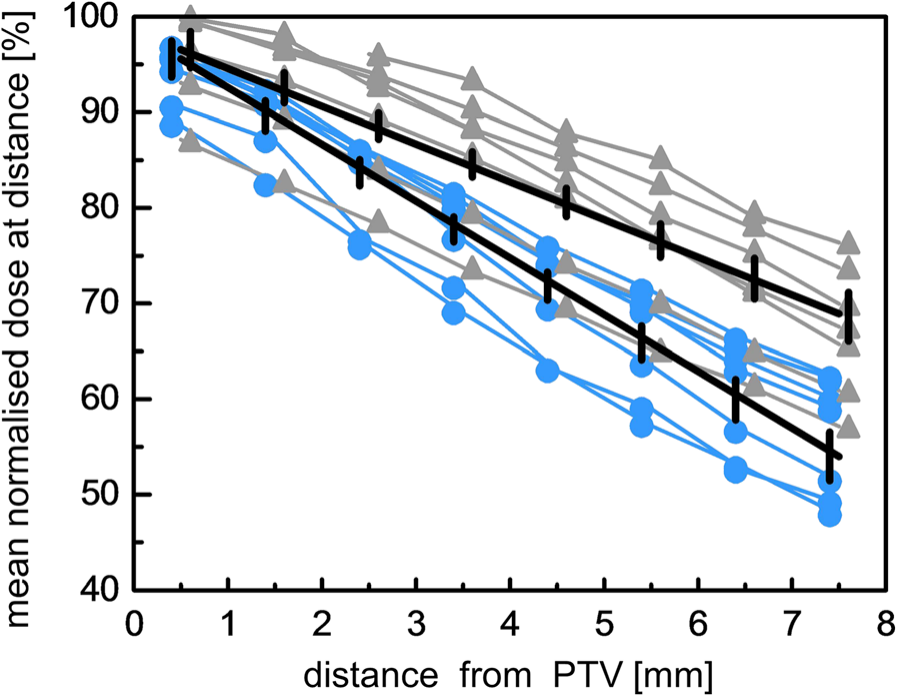
**Dose fall-off.** Dose fall-off outside planning target volume for helical tomotherapy (HT) (blue) and intensity modulated proton therapy (IMPT) (grey). Mean doses in adjacent shells of 1 mm width around the planning target volume (PTV) within the respective patient’s body are given normalized to the prescribed dose. The slopes of the average dose fall-off differed between HT and IMPT (p< 0.0001). In addition, the 95% confidence intervals for the predicted dose values at a given distance are indicated by vertical bars.

Figure 3 illustrates the dose fall-off outside PTV1 for the 7 patients for HT and IMPT normalized by the prescribed dose. Mean doses are shown in adjacent shells of 1 mm width around the PTV1 within the respective patient’s body. The dose fall-offs were adequately described by a linear dependence on distance from PTV. A quadratic term did not become significant. The slopes were significantly steeper for HT than for IMPT (−5.94 ± 0.26%/mm vs. -3.99 ± 0.26%/mm, p< 0.0001, F-test). To give an impression of the steepest dose fall off achieved around the PTV1, the D90 values in the adjacent 1 mm shells were analyzed. Again, gradients with HT were significantly steeper than with IMPT (−8.69 ± 0.36%/mm vs. -7.33 ± 0.36%/mm, p< 0.0001, F-test).

### Normal tissue exposure

Spinal cord or brainstem exposures were compared by D2 and Dmax or D_1cm_^3^ and Dmax as the respective end points (Table 1). The paired dose differences between IMPT and HT normalized to the prescription dose were not significantly different from 0 Gy_RBE_ over all patients and all 4 parameters related to spinal cord and brainstem exposure (average difference, -1.6 ± 1.0 Gy_RBE_, p=0.073, signed rank). The normalized dose differences between IMPT and HT were smaller for the parameters D2 or D_1cm_^3^ (average ΔD2 or ΔD_1cm_^3^ = −3.7%) as for D_max_ (average ΔD_max_ = +0.4%). All plans fulfilled the clinical criteria, i.e. life time accumulated D_max_ to the spinal cord and brain stem had to remain below 60 Gy_RBE_ and 64 Gy_RBE_, respectively.

With respect to the ipsilateral optic nerve and the chiasm, no significant D_max_ differences were seen between the IMPT and HT plans (Table 1). The average ΔD_max_ between both methods was 0.5 ± 2.4% of the prescription dose for the 7 patients (p=0.89).

Ipsilateral temporal lobe and cerebellum exposures were evaluated by Dmean, and one parameter related to the hot spots in this structure, either D_1cm_^3^ or D_5cm_^3^. The normalized Dmean differences between IMPT and HT for ipsilateral temporal lobes were not different from 0% (average ΔD_mean_ = 0.5 ± 2.8%; p=0.58, signed rank test), neither was the D_1cm_^3^ (average ΔD_1cm_^3^ = 0.6 ± 2.0%; p=0.94). The average ΔD_mean_ for the cerebellum was lower with IMPT (−9.1 ± 2.5%, p=0.02), the average ΔD_1cm_^3^ for the patients of this study was not (1.0 ± 3.5%, p=0.99).

In addition, the average Dmean difference of the parotid gland exposure was −4.5 ± 3.4% (p=0.22). Mean body dose by IMPT was lower in 6 patients and equal in 1 patient compared with HT, on average by 1.6 ± 0.4% of the prescribed dose (p=0.03).

## Discussion

Considering target coverage, safety margins alone cannot ensure dose coverage of the CTV for IMPT plans with multiple fields and high in-field dose gradients. Deviations in the position of these dose gradients in the patient from field to field due to set-up errors or range uncertainties can result in under- or overdosage inside the PTV [15,16]. Therefore robust planning was employed to optimize the dose distribution simultaneously for multiple scenarios mimicking set-up errors and range uncertainties and minimizing the penalty of the worst case scenario [12]. Robust optimization can lead to treatment plans considerably less sensitive to set-up errors and range uncertainties than IMPT plans optimized using PTV-based conventional methods. In addition, robust IMPT optimization was able to result in higher conformity than margin-based IMPT optimization methods [17]. HT and IMPT with small pencil beams had similar target coverage and robustness against set-up errors within 2 mm. Highly constrained IMPT plans showed some residual dependence of brainstem D2 on range uncertainties. Consideration of a set-up error of 2 mm seems adequate for small targets in the head and neck region with daily online navigation [18]. While on average the HT plans in our comparison showed a higher conformity around the CTV in the high dose region down to 50% of the prescribed dose or within 7.5 mm around the PTV, IMPT plans had a reduced low dose bath at isodoses below 50%. Similarly, Seco in 2011 reported of larger high dose regions with passively scattered protons in comparison to photon stereotactic body radiotherapy for smaller stage I lung cancer [19].

IMPT for cancers of the head and neck region was investigated in previous studies in comparison to fixed field or rotational IMXT [20-25]. These studies used a PTV margin based concept to consider set-up errors for proton therapy and none used robust optimization. All of these studies used 2–3 field IMPT plans. Four of these studies employed fixed-field IMXT, four HT for comparison with IMPT [20,23,25,26]. While all of the studies using fixed-field IMXT found that IMPT had a greater potential to spare adjacent normal tissues, the studies using HT for comparison found similar conformity and normal tissue exposure. These studies included only 1, 1, 3, and 6 patients, respectively. The target volumes in the latter studies, however, tended to be larger than in the present study. The target conformity of the HT plans in the present study compared well with the photon plans in most of the above studies [21,24,25], and the present study extends the comparison of IMPT and HT to smaller complex target volumes and nearby organs at risk, using robust IMPT optimization. Four of the above studies gave the ICRU conformity index at the 95% isodose (CI95%) (ICRU reports 50 and 62) around the PTV for their IMPT plans [21,22,24,25]. The mean CI95% ranged from 1.02 - 1.40. In the present study, the average CI95% was 1.29 ± 0.24 with IMPT, a value in the middle range of the above studies. But the median target volume in this study was smaller than in most of the above studies. It is well known that the CI decreases and therefore conformity improves for a considered radiotherapy method with increasing target volumes [27]. The robustness of the D95 for CTV-coverage against set-up errors was found similar for robust IMPT and HT. While adequate brain, brainstem and spinal cord sparing could be achieved with IMPT at the level preset by the HT plans, temporal lobe doses and optic nerve doses were not substantially different on average. D_mean_ but not Bitte D1cm3 wie auf der vorhergehenden Seite: cm tief, 3 hoch gesetzt. to the cerebellum and the mean body dose were lower on average with IMPT for the patients of this study. Comparing HT with other rotational IMRT methods such as volumetric modulated arc therapy showed a similar conformity that was superior to static field IMRT so that the results here in comparison to HT can be generalized to rotational IMRT methods with photons [28].

In the re-irradiation situation, it can be difficult to weight the better high dose conformity of HT against the reduced low dose exposure by IMPT. Several of the severe side effects that can be consequence of re-irradiation, such as trismus, osteoradionecrosis, subcutaneous fibrosis, brain stem necrosis, and cranial neuropathy and carotid rupture seem to be related rather to the volume of the high dose region than the low dose bath of radiotherapy below the 50% isodose [13,29,30]. If steep dose gradients in the near vicinity of the target volume are of dominant concern, rotational IMRT is a particularly good radiotherapy solution. The known issue of difficult skin sparing with IMPT, relevant for superficially located target volumes such as head and neck lymph node regions, was not considered in the comparison of the methods.

The conformity of both methods, rotational IMXT and IMPT, has potential to be improved in the future. Avenues for possible further improvements with IMPT for shallow depth head and neck carcinomas include using smaller pencil beam width especially at low energies allowing for treatments without range shifters, smaller air gaps, and further refinement of robust optimization using e.g. contour-related spot placing and spacing. Conformity with rotational IMXT can be improved for instance by smaller jaw width, smaller pitch and higher modulation factor in the case of helical therapy and the use of small penumbra micromultileaf collimators, use of noncoplanar, multiple arcs in the case of C-arm linear accelerators, or the use of multiple degrees of freedom robotic systems for beam delivery.

## Conclusion

Potentials of intensity modulated proton therapy plans using robust optimization were investigated under set-up and range uncertainty conditions. While HT showed on average a higher conformity down to the 50% isodose and steeper dose gradients within 7.5 mm outside the PTV in the high dose region, IMPT had a reduced low dose bath. Because neither of the two methods, IMPT or HT, was found uniformly better in terms of target coverage and organs at risk sparing, comparative planning is recommended for the individual patient in the clinical situation of retreatment of recurrent head and neck cancer, provided both methods are available.

## Competing interests

Parts of this work were supported by Grant No. STU-151/9-1 from the German Research Foundation (Deutsche Forschungsgemeinschaft). The authors declare that they have no competing interests.

## Authors' contributions

MS carried out the plan comparison, performed statistical analysis and drafted the manuscript. He also contributed to the optimizations of both, helical tomotherapy and proton therapy plans. AK performed the robust optimization and evaluation of the IMPT plans. He reviewed and edited draft versions of the manuscript. JAJ and CP conducted clinical treatment of the patients, generated initial volumes of interest, and approved clinical helical tomotherapy plans. SL performed the helical tomotherapy optimization and performed dosimetric analysis of both, tomotherapy and IMPT plans. JF was involved in improving the proton therapy optimization approaches and in the design of the proton therapy planning system. He also participated in concept discussions for developing the investigative aims and critically reviewed the draft manuscript. All authors read and approved the final manuscript.
